# Perceptions of doctors to adverse drug reaction reporting in a teaching hospital in Lagos, Nigeria

**DOI:** 10.1186/1472-6904-9-14

**Published:** 2009-08-11

**Authors:** Kazeem A Oshikoya, Jacob O Awobusuyi

**Affiliations:** 1 Pharmacology Department, Lagos State University College of Medicine, P.M.B 21266, Ikeja, Lagos, Nigeria; 2 Paediatrics Department, Lagos State University Teaching Hospital, Ikeja, Lagos, Nigeria; 3 Medicine Department, Lagos State University College of Medicine, P.M.B 21266, Ikeja, Lagos, Nigeria

## Abstract

**Background:**

Spontaneous adverse drug reaction (ADR) reporting is the cornerstone of pharmacovigilance. ADR reporting with Yellow Cards has tremendously improved pharmacovigilance of drugs in many developed countries and its use is advocated by the World Health Organization (WHO). This study was aimed at investigating the knowledge and attitude of doctors in a teaching hospital in Lagos, Nigeria on spontaneous ADR reporting and to suggest possible ways of improving this method of reporting.

**Methods:**

A total of 120 doctors working at the Lagos State University Teaching Hospital (LASUTH), in Nigeria were evaluated with a questionnaire for their knowledge and attitudes to ADR reporting. The questionnaire sought the demographics of the doctors, their knowledge and attitudes to ADR reporting, the factors that they perceived may influence ADR reporting, and their levels of education and training on ADR reporting. Provision was also made for suggestions on the possible ways to improve ADR reporting.

**Results:**

The response rate was 82.5%. A majority of the respondents (89, 89.9%) considered doctors as the most qualified health professionals to report ADRs. Forty (40.4%) of the respondents knew about the existence of National Pharmacovigilance Centre (NPC) in Nigeria. Thirty-two (32.3%) respondents were aware of the Yellow Card reporting scheme but only two had ever reported ADRs to the NPC. About half (48.5%) of the respondents felt that all serious ADRs could be identified after drug marketing. There was a significant difference between the proportion of respondents who felt that ADR reporting should be either compulsory or voluntary (χ^2 ^= 38.9, *P *< 0.001). ADR reporting was encouraged if the reaction was serious (77, 77.8%) and unusual (70, 70.7%). Education and training was the most recognised means of improving ADR reporting.

**Conclusion:**

The knowledge of ADRs and how to report them are inadequate among doctors working in a teaching hospital in Lagos, Nigeria. More awareness should be created on the Yellow Card reporting scheme. Continuous medical education, training and integration of ADR reporting into the clinical activities of the doctors would likely improve reporting.

## Background

Adverse drug reactions (ADRs) are global problems of major concern. They affect both children and adults with varying magnitudes, causing both morbidity and mortality [1-4]. In addition to the human costs, ADRs have a major impact on public health by imposing a considerable economic burden on the society and the already-stretched health-care systems [5,6]. Post marketing surveillance of drugs is very important in analysing and managing the risks associated with drugs once they are available for the use of the general population. Spontaneous reporting has contributed significantly to successful pharmacovigilance. The contribution of health professionals, in this regard, to ADRs databases is enormously significant and has encouraged ongoing ascertainment of the benefit-risk ratio of some drugs [7,8] as well as contributed to signal detection of unsuspected and unusual ADRs previously undetected during the initial evaluation of a drug [9,10]. In spite of these benefits, under-reporting remains a major draw-back of spontaneous reporting [10,11]. It is estimated that only 6–10% of all ADRs are reported [12,13]. This high rate of under-reporting can delay signal detection and consequently impart negatively on the public health.

Many factors are associated with ADRs under-reporting among health professionals. These factors have been broadly classified as personal and professional characteristics of health carers, and their knowledge and attitudes to reporting [11]. Inman [14] has summarized these factors as the 'seven deadly sins'. His description of the 'sins' include: attitudes relating to professional activities (*financial incentives*: rewards for reporting; *legal aspects*: fear of litigation or enquiry into prescribing costs; and ambition to compile or publish a personal case series) and problems associated with ADR-related knowledge and attitudes (*complacency*: the belief that very serious ADRs are well documented by the time a drug is marketed; *diffidence*: the belief that reporting an ADR would only be done if there was certainty that it was related to the use of a particular drug; *indifference*: the belief that the single case an individual doctor might observe could not contribute to medical knowledge; and *ignorance*: the believe that it is only necessary to report serious or unexpected ADRs), and excuses made by professionals (*lethargy*: the procrastination and disinterestedness in reporting or lack of time to find a report card and other excuses). Lopez-Gonzalez et al [11], in their review of determinants of ADRs under-reporting from the global perspective, have shown that three of the seven 'sins' proposed by Inman that are associated with professional activity (financial incentives, fear and ambition to publish) seem to contribute less significantly to under-reporting. Insecurity (the belief that it is nearly impossible to determine whether or not a medicine is responsible for a particular ADR) is another factor associated with under-reporting [11] but was not proposed by Inman. It therefore appears that factors that promote under-reporting may vary from one country to another.

The determinants of ADRs under-reporting have not been extensively studied in Africa. Only two studies have analysed these factors in African countries [15,16]. A previous study from Nigeria has indicated inadequate knowledge of resident doctors about ADRs [17]. However, the study excluded other cadres of doctors, it did not evaluate attitudes that were associated with under-reporting and did not assess the in-depth knowledge of the doctors about ADRs reporting.

This study was therefore aimed at investigating the knowledge and attitudes of doctors to ADR reporting in a teaching hospital in Lagos, Nigeria and to suggest possible ways of improving spontaneous reporting based on our findings.

## Methods

### Setting

The study was conducted at the Lagos State University Teaching Hospital (LASUTH) in Nigeria. This is one of the two teaching hospitals in Lagos that provide both medical and dental care in the entire specialty to over 15 million populations in Lagos State. Altogether, over a hundred doctors; consisting of interns, medical officers, residents and consultants work in the hospital. Medical and dental care services at the LASUTH are free of charge and this enables the hospital to enjoy a very high patronage. LASUTH has an ADR Monitoring Committee that is charged with the responsibility of reviewing all suspected cases of ADRs and forwarding the list of confirmed cases to the National Pharmacovigilance Centre (NPC).

### Design and study population

This is a cross-sectional study involving 120 doctors who were surveyed with a questionnaire (Additional file 1). The questionnaire was adapted from the previous studies that assessed attitudes of medical practitioners and pharmacists to ADR reporting in the United Kingdom [18-21] with a little modifications to suit the Nigerian environment. The questionnaire was structured to obtain the demographics of the doctors, information about their knowledge of ADR reporting, attitudes to reporting, factors that they perceived may influence reporting, and their levels of education and training on ADR reporting. Provision was also made for suggestions on possible ways to improve ADR reporting. A list of hypothetical cases of suspected ADRs with the culprit drugs (palpitation to artemether/lumefantrine (coartem^®^), jaundice to frusemide, hiccups to enalapril, fixed drug eruption to co-trimoxazole or sulphadoxine/pyrimethamine, gastrointestinal bleeding to diclofenac and thrombocytopenia to heparin) was provided in the questionnaire to assess the practical and in-depth knowledge of ADR reporting by the doctors.

### Data collection

The questionnaire was validated through a pilot study of 20 randomly selected doctors (both medical and dental) from another teaching hospital, the Lagos University Teaching Hospital (LUTH), Idiaraba.

The questionnaire was distributed through the various Heads of Department and was allowed to stay with the doctors for 4 weeks so as to allow them enough time to attend to the questions. While retrieving the questionnaire the same copy was re-administered to those who could not produce the previous copy given to them. This is to encourage non-respondents to participate in the study. The re-administered questionnaire was filled in and returned the same day unless the doctor was no longer interested in the study.

The Ethics committee of LASUTH approved the study.

### Statistical analysis

Data were analysed using SPSS version 13. Results are presented as mean ± standard deviation for quantitative variables, median with inter-quartile range (IQR) for time related variables and numbers with percentages or graphic presentations for categorical variables. The relationship between the position of the respondents and their general knowledge of ADRs or their in depth knowledge of the illustrated hypothetical cases was determined by using a chi-square at *P *< 0.05 significant level.

## Results

### Demographics

Of the 120 questionnaires distributed, 99 were duly filled and returned thus giving a response rate of 82.5%. The characteristic features of the respondents are shown in Table 1.

**Table 1:** The demographics and characteristic features of the respondents

**Characteristics**	**Values**
Median age (years)	36 (IQR 32–40)

Median year of first degree qualification (years)	10 (IQR 4–15)

Median year of practice in a teaching hospital (years)	3 (IQR 1.5–7.0)

Male: female ratio	59: 40

Cadre	

*Interns*	30 (30.3%)

*Junior residents*	29 (29.3%)

*Consultants*	22 (22.2%)

*Senior residents*	18 (18.2%)

Country of undergraduate education	

*Nigeria*	84 (84.8%)

*Abroad*	15 (15.2%)

Additional qualifications	37 (37.4%)

### Doctors' knowledge of ADR reporting scheme and pharmacovigilance

The vast majority of the respondents (89, 89.9%) knew that, as doctors, they could report ADRs. Dentists (61, 61.6%), nurses (63, 63.6%) and pharmacists (63, 63.6%) were also considered capable of reporting ADRs. Individual persons (42, 42.5%) and physiotherapists (14, 14.1%) were considered the least important people to report ADRs. About half of the respondents (51, 51.5%) were aware of the existence of NPC in Nigeria among whom 20 (39.2%) correctly identified Abuja as the office. Less than a half (32, 32.3%) of the respondents was aware of the Yellow Card reporting scheme in Nigeria among whom only two had ever reported ADRs with a Yellow Card submitted to the NPC. The reporting was done only once by each of the respondents. The purposes of Yellow Card reporting scheme were incorrectly identified by the respondents as to identify safe drugs (35, 35.4%) and to calculate incidence of ADRs (25, 25.3%). Contrarily, the purposes of Yellow Card scheme were correctly identified by the respondents as to identify previously unrecognised reactions (44, 44.4%), to serve as a source of information about the characteristics of reactions (34, 34.3%) and to compare the adverse effects of drugs within the same therapeutic class (30, 30.3%). Most of the respondents knew that all ADRs should be reported to newly marketed drugs (92, 92.9%), and serious reactions should be reported for established products (88, 88.9%). When compared with the position/level of the respondents, there was no significant difference in reporting ADRs to newly marketed drugs (χ^2 ^= 3.7, *P *= 0.49) and serious reactions to established products (χ^2 ^= 5.1, *P *= 0.28). When compared with the responses for serious reactions to established products, a significantly higher number of the respondents knew that all reactions should be reported for over the counter (OTC) drugs (65/99; χ^2 ^= 4.6, *P *= 0.03) and topical agents (63/99; χ^2 ^= 2.25, *P *= 0.04), but the difference was not significant for vaccines (84/99; χ^2 ^= 1.4, *P *= 0.27), herbal medicines (68/99; χ^2 ^= 0.14, *P *= 0.72), antibiotics (89/99; χ^2 ^= 0.01, *P *= 0.93), antimalarials (66/99; χ^2 ^= 0.1, *P *= 0.75) reporting.

### Attitudes to reporting ADRs

Sixty-four respondents (64.6%) felt that ADR reporting was a professional obligation, 63 (63.6%) felt that all serious ADRs could be identified after a drug had been marketed and that one report made no difference to the Yellow Card reporting scheme. About half (52, 52.5%) of the respondents opined that ADR reporting should be compulsory, 36 (36.4%) stated that ADR reporting should be voluntary and 11 (11.1%) were either unsure or not responding. There was a significant difference between the proportion of respondents that felt ADR reporting should be either compulsory or voluntary (χ^2 ^= 38.9, *P *< 0.001). A very few respondents felt ADR reporters should be remunerated (22, 22.2%), have their identity hidden (18, 18.2%) and also the identity of the prescribers hidden (4). The two respondents that had alerted NPC of ADR with a Yellow Card were clear of what should be reported, however they both found the form too complex to fill.

### Factors influencing ADR reporting

The respondents were encouraged to report ADRs if the reaction was serious (77, 77.8%) and unusual (70, 70.7%) in nature. Other factors that would influence ADR reporting include if the reaction was to a new product (58, 58.6%), certainly that the reaction was truly an ADR (45, 45.5%), and if the reaction was well recognised for a particular drug (46, 46.5%). Contrarily, those factors that would discourage the respondents from reporting ADRs are listed in Table 2. The fear of the report being wrong (47, 47.5%) was the most discouraging factor.

**Table 2:** Factors that may discourage doctors from reporting adverse drug reaction

**Factor**^a^	**Agree**	**Disagree**
Concern that the report may be wrong	47 (47.5%)	52 (52.5%)

Lack of time to fill in a report and a single unreported case may not affect ADR database	37 (37.4%)	62 (62.6%)

Level of clinical knowledge makes it difficult to decide whether or not an ADR has occurred	36 (36.4%)	63 (63.6%)

Lack of time to actively look for an ADR while at work	33 (33.3%)	66 (66.7%)

Don't feel the need to report well recognised actions	27 (27.2%)	72 (72.2%)

The absence of fee for reporting	18 (18.2%)	81 (81.8%)

Concern that a report will generate an extra work	15 (15.2%)	84 (84.8%)

Fear of the negative impact the report may have on the company that produced or marketed the drug	15 (15.2%)	84 (84.8%)

Lack of confidence in discussing the ADR with other colleagues	12 (12.1%)	87 (87.9%)

^a ^Number of doctors responding (n = 99).

From the list of hypothetical cases of ADRs illustrated to the respondents, only five examples listed in Table 3 were considered reportable to the NPC. Palpitation to coartem^® ^(58/98, 59.2%), skin rashes to roxithromycin (54/98, 55.1%) and jaundice to frusemide (53/98, 54.1%) would be reported more frequently. A significantly higher proportion of the respondents would report fixed drug eruption to co-trimoxazole and sulphadoxine/pyrimethamine than hiccup to enalapril (χ^2 ^= 12.5, *P *= 0.02). There was no significant difference in the proportion of respondents that would report fixed drug eruption to co-trimoxazole and sulphadoxine/pyrimethamine and headache to isordil dinitrate (χ^2 ^= 2.2, *P *= 0.68).

**Table 3:** Proportion of respondents that would report hypothetical cases of ADRs in line with the National Pharmacovigilance Centre reporting criteria

**Reaction**^a^	**Yes**	**No**	**Don't know**
Palpitation with Coartem^® ^(Yes)^b^	58 (59.2%)	20 (20.4%)	20 (20.4%)

Skin rashes with roxithromycin (Yes)^b^	54 (55.1%)	26 (26.5%)	18 (18.4%)

Jaundice with frusemide (Yes)^b^	53 (54.1%)	21 (21.4%)	24 (24.5%)

Thrombocytopenia with heparin (Yes)^b^	39 (39.8%)	36 (36.7%)	23 (23.5%)

Hiccup with enalapril (No)^b^	36 (36.7%)	29 (29.6%)	33 (33.7%)

Gastrointestinal bleed with diclofenac (Yes)^b^	35 (35.7%)	41 (41.8%)	22 (22.4%)

Headache with isordil dinitrate (No)^b^	33 (33.7%)	41 (41.8%)	22 (22.4%)

^a ^Number of doctors responding (n = 98), ^b ^Response refer to standard NPC reporting in Nigeria.

### Education and training on ADRs

Only one respondent had received training on how to report ADR with a Yellow Card. However, the respondent did not mention where the training was received. The majority of the respondents (98, 98.9%) are willing to undergo training on how to recognise ADRs and how to report them with a Yellow Card.

### Improving ADRs reporting

The various methods suggested by the respondents to improve ADR reporting are presented in Table 4. Continuous medical education, training and refresher courses (94, 95.9%) were the methods mostly recommended. Leaving the Yellow Card on the ward for easy accessibility (6) was considered the least important method.

**Table 4:** Suggested methods of improving ADRs reporting

**Methods**	**Frequency (n = 98)**	**Percentage (%)**
Continuous medical education, training and refresher study	94	95.9

Instituting and encouraging feedback between patients prescribers and dispensers of drugs	69	70.4

Reminders and increased awareness from the ADR Monitoring Committee	67	68.4

Increasing awareness among other professionals that they could report ADRs	62	63.3

Increased collaboration with other healthcare professionals	58	59.2

More publicity about reporting scheme in local journals	56	57.1

Encouragement from the ADR Monitoring Committee and various head of departments	49	50.0

Having an ADR specialist in every department	46	46.9

Encouraging on-line or telephone reporting	45	45.9

Alerting all outpatients to watch out for possible ADR when prescribing new drugs	44	44.4

Remuneration for every reported case of ADR	28	28.6

Spending more time on the wards with patients	26	26.5

Making reporting a professional obligation	25	25.5

Incentives to every outpatient that report ADR	21	21.4

Leaving Yellow Cards on the ward for easy accessibility	6	6.1

## Discussion

This study has shown inadequate knowledge of doctors about ADRs and reporting similar to the previous reports among resident doctors in Nigeria [17] and doctors in many countries across Europe [18,22-24], America [25,26] and Asia [27,28]. Perhaps, the undergraduate training in pharmacovigilance and medicine risk perceptions may be either insufficient or improperly delivered to prepare the doctors for the task of ADR monitoring and reporting in their future career.

Spontaneous ADR reporting by other health professionals and individuals is practiced in many countries [29-31] and it is recommended by the NPC [32] but not recognised by the respondents. This is reflected in their low percentages that considered individuals and physiotherapists qualified to report ADRs. A significant number of the respondents were not aware of the existence of a national pharmacovigilance centre in Nigeria and amongst those who were aware, only 39.2% were able to correctly identify Abuja as the office. Lack of knowledge of where ADRs should be reported would automatically affect reporting, therefore, awareness programmes; through publicity, would appear necessary to improve ADR reporting in Nigeria.

The general lack of awareness of ADR reporting system in Nigeria was reflected by the 63.4% of the respondents who did not know about the existence of a Yellow Card reporting scheme coupled with the fact that only two respondents had ever reported ADRs with a Yellow Card. This proportion is rather very low when compared to a similar reporting scheme among doctors in the United Kingdom [19], America [25], Netherland [33], Spain [34], China [28] and India [35]. The differences in the reporting rates may be attributed to the priority, attention and commitment given to pharmacovigilance by the government of these countries. Such attitudes need to be emulated by the Nigerian government.

When we compared the factors that may influence reporting by the respondents with those reported by Lopez-Gonzalez et al [11], the results were similar. Our study has shown that, like most countries around the world, ignorance (not feeling the need to report well recognised reaction), diffidence (concern that the ADR report may be wrong) and indifference (lack of time to fill in a report and a single unreported case may not affect ADR database) (Table 2) would significantly influence ADR-reporting among the doctors working in a Nigerian teaching hospital. However, complacency, fear, financial incentives and bureaucracy involved in filling in the Yellow Card would have a little influence on the respondents to report ADRs. Therefore ADR under-reporting in Nigeria appears to be associated more with knowledge gaps and attitudes of the doctors rather than with personal and professional characteristics reported in other studies [18,19,36,37]. Previous studies have shown that distribution and availability of Yellow Cards to the doctors increase ADRs reporting [22,38] but unfortunately, a very high proportion of the respondents did not consider this as an important means of improving reporting.

Most of the respondents were willing to report reactions to newly marketed drugs and serious reactions to established products because they perceived post-marketing surveillance as an important part of pharmacovigilance. Globally, vaccines and antibiotics are among the leading causes of ADRs, especially in children [39-42]. In addition to antibiotics, herbal medicines and antimalarial drugs have contributed significantly to ADRs in Nigeria [39]; this may therefore explain the higher number of respondents who would report serious ADRs to these drugs when compared with OTC drugs and topical agents. Post marketing drug surveillance encouraged ADR reporting if reaction was serious and unusual in nature. These encouraging factors were recognised by over 70% of the respondents. The NPC is known not to receive reports of proven reactions [32]. The respondents may probably not be aware of this as 46.5% of them would report a well recognised reaction to a particular drug.

Anxiety of respondents not to appear incompetent or become subject of ridicule may cause them to want to report only ADRs that they consider certainly were caused by a drug. Such fear may probably explain the 45.5% respondents with this response which is a consistent finding in other studies [18,21]. The need to allay this fear, through sensitisation and pharmacovigilance education by NAFDAC, cannot be over-emphasized.

The five hypothetical examples of ADRs reportable to NPC were recognised by 30% to 50% of the respondents. These values are rather low. Palpitation with the use of coartem, skin rashes to roxithromycin, and jaundice to frusemide were more recognised than thrombocytopenia to heparin and gastrointestinal bleeding to diclofenac as unusual reactions. The NAFDAC may need to formulate a guideline for health professionals to improve recognition of unusual ADRs.

Educational intervention has been shown to improve ADR reporting in Portugal [43] and Rhode Island in the USA [36]. Education and training on spontaneous ADR reporting and how to use the Yellow Card is very necessary among the doctors since only one respondent had ever received such training. Almost all the respondents showed interest in education and training. Continuous Medical Education, training and refresher courses were the most cited means of improving ADR reporting. This certainly shows that the doctors are willing to improve their knowledge of ADR reporting and increase their participation in the Yellow Card reporting scheme if education and awareness on the reporting scheme is instituted in the hospital. The other methods recommended by the respondents such as instituting and encouraging feedback between patients, prescribers and dispensers of drugs, receiving reminders and increased awareness from ADR Monitoring Committee, and increasing the awareness of other healthcare professionals on reporting ADRs are very important and can certainly be considered as examples of a good practice that should be instituted in the hospital. Most of the factors considered unimportant to improve ADR reporting (Table 4) had been implemented in the developed countries and have yielded good results [14,18,19,44]. Efforts should be made at implementing these methods in the teaching hospital.

The objective of this study was to evaluate the attitudes and knowledge of doctors in a teaching hospital in Lagos, Nigeria to spontaneous ADR reporting. However, this study was limited by not comparing the attitudes and knowledge of the doctors with their years of practice and position/level. This is likely to be addressed in our future studies. It would be logical to extend this study to other teaching hospitals in Nigeria to enable us generalise our findings.

## Conclusion

There are gaps between knowledge and ADRs reporting among doctors working in a teaching hospital in Lagos, Nigeria. These gaps need to be filled by improved training in pharmacovigilance and risk perceptions of drugs. It may take the doctors some time to fully accept ADR reporting as a role if continuous medical education, reminders and awareness on the Yellow Card reporting scheme are not instituted in the hospital. Attitudinal and cultural changes, whereby ADR reporting is seen as an integral part of the clinical activities of the doctors, are very necessary for a long term improvement of ADR reporting.

## Abbreviations

ADR: Adverse drug reaction; WHO: World Health Organisation; LASUTH: Lagos State University Teaching Hospital; NPC: National Pharmacovigilance Centre; NAFDAC: National Agency for Food and Drug Administration; IQR: Interquartile Range and Control; OTC: Over The Counter.

## Competing interests

The authors are members of adverse drug reaction motoring committee of the Lagos State University Teaching Hospital, Ikeja, where the study was conducted.

## Authors' contributions

KAO and JOA conceived the study, designed the study and questionnaire, and participated in distributing the questionnaires. KAO performed the statistical analysis and was reviewed by JOA. Both authors drafted the manuscript and approved the final version.

## Pre-publication history

The pre-publication history for this paper can be accessed here:



## Supplementary Material

Additional file 1 **Questionnaire for evaluating physicians' knowledge and attitudes to ADR reporting**. The questionnaire sought the demographics of the doctors, their knowledge and attitudes to ADR reporting, the factors that they perceived may influence ADR reporting, and their levels of education and training on ADR reporting. Click here for file
