# Supplementary material for: Perceptions of doctors to adverse drug reaction reporting in a teaching hospital in Lagos, Nigeria
Source: BMC Clin Pharmacol. 2009 Aug 11;9:14. doi: 10.1186/1472-6904-9-14 (PMC2731723; doi:10.1186/1472-6904-9-14)
Supplement: Additional file 1 — Questionnaire for evaluating physicians' knowledge and attitudes to ADR reporting. The questionnaire sought the demographics of the doctors, their knowledge and attitudes to ADR reporting, the factors that they perceived may influence ADR reporting, and their levels of education and training on ADR reporting. [file 1472-6904-9-14-S1.doc]

**Perceptions of adverse drug reaction reporting by doctors working in a teaching hospital in Lagos, Nigeria**

The adverse drug reaction (ADR) monitoring committee of the Lagos State University Teaching Hospital (LASUTH) is conducting a study to investigate the knowledge and attitudes of the doctors at the LASUTH to ADR reporting and to suggest the possible ways of improving spontaneous reporting based on our findings.

We need your maximum co-operation to fill the questionnaire and return them in time. This study is purely for academic purposes and self-sponsored. All your responses shall be treated with utmost confidentiality.

**Demographics**

1. How are old are you? ...................................................................... (please specify)
2. Your gender is? (a) male (b) female
3. Your department is? (a) medicine (b) surgery (c) obstetrics and gynecology (d) pediatrics (e) family medicine (f) accident and emergency (g) ophthalmology (h) radio diagnosis (i) radiotherapy and oncology (j) anesthesia (k) dentistry (l) others (specify)…………………………………………………………………………….
4. Position/level (a) consultant (b) senior registrar (c) junior registrar (d) chief medical officer (e) principal medical officer (f) senior medical officer (h) medical officer (i) house officer (j) others (specify)……………………………………….
5. Year of undergraduate qualification in medicine is ……………… (please specify)
6. Years of practice in a teaching hospital is ………………………... (please specify)
7. Country of undergraduate MBBS training is …………………….. (please specify)
8. Country where additional qualification was obtained, if any? ……………(specify)

**Knowledge of ADRs**

1. Which of the following health professionals are qualified to report adverse reaction to drugs? (a) medical doctors (b) dental doctors (c) nurses (d) pharmacists (e) physiotherapist (f) individual persons
2. Are you aware of the existence of a National Pharmacovigilance Centre (NPC) in Nigeria? (a) yes (b) no
3. If the above is yes, where is the NPC office located? …………… (please specify)
4. Are you aware of the yellow card reporting scheme for reporting ADRs in Nigeria? (a) yes (b) no
5. If the above is yes, have you ever submitted a yellow card to the NPC? (a) yes (b) no
6. Are you aware of the ADR Monitoring Committee at LASUTH? (a) yes (b) no
7. If the above is yes, how many times have you alerted them of an ADR? ……………………………………………………………… (please specify)
8. All ADRs should be reported for newly marketed agents? (a) yes (b) no
9. Serious reactions should be reported for established products? (a) yes (b) no
10. The following agents should be reported for ADRs (a) vaccines (b) herbal and complementary medicines (c) over the counter (OTC) drugs (d) antibiotics (e) antimalarials (f) topical agents
11. All serious ADRs could be identified to a drug after it has been marketed (a) yes (b) no
12. The purpose of yellow card reporting scheme include (a) to identify safe drugs (b) to calculate incidence of ADRs (c) to identify predisposing factors to ADRs (d) to identify previously unrecognized ADRs (e) to serve as a source of information about the characteristics of the adverse reaction (f) for comparison of Adverse effects of drugs within the same therapeutic class.

**Attitudes to reporting ADRs**

1. Factors that may encourage you to report an ADR include (a) if the reaction was serious (b) if the reaction was unusual (c) if the reaction was to a new product (d) if the reaction was certainly an ADR (e) if the reaction was well recognized for a particular drug
2. Which of the following factors may discourage from reporting an ADR? (a) concern that the report may be wrong (b) lack of time to fill-in a report and a single unreported case may not affect ADR database (c) non-remuneration for reporting (d) concern that reporting may generate extra work (e) lack of time to actively look for ADRs while at work (f) level of clinical knowledge makes it difficult to decide whether or not an ADR has occurred (g) lack of confidence to discuss the ADR with other colleagues (h) do not feel the need to report a recognized ADR (i) fear of the negative impact the report may have on the company that produced or marketed the drug.
3. ADR reporting is a professional obligation (a) yes (b) no (c) don’t know
4. Reporting of only one ADR makes no significant contribution to the yellow card reporting scheme (a) yes (b) no (c) don’t know
5. If you have ever filled the yellow card for ADR reporting, was the information on it very clear to you about what to report? (a) yes (b) no
6. The yellow card is too complex to fill (a) yes (b) no
7. ADR reporting should be (a) compulsory (b) voluntary (c) remunerated (d) hide the identity of the prescriber (e) hide the identity of the reporter

**Reporting of hypothetical cases of ADRs**

Which of the following suspected ADRs would you report to the NPC?

1. Jaundice following the use of frusemide (a) yes (b) no (c) don’t know
2. Skin rashes to roxithromycin use (a) yes (b) no (c) don’t know
3. Headache following the use of isordil dintrate (a) yes (b) no (c) don’t know
4. Palpitation following the use of coartem (a) yes (b) no (c) don’t know
5. Hiccups following the use of enalapril (a) yes (b) no (c) don’t know
6. Fixed drug eruption following the use of co-trimoxazole or sulphadoxime/pyrimethamine (a) yes (b) no (c) don’t know
7. Thrombocytopenia following the use of heparin (a) yes (b) no (c) don’t know
8. GIT bleeding following the use of diclofenac sodium (a) yes (b) no (c) don’t know

**Education and training/improving ADR reporting**

1. Have you ever been trained on how to report ADR with a yellow card? (a) yes (b) no
2. If the above is yes, where were you trained? (please specify)……………...………
3. Suggest possible ways of improving ADR reporting (please list as many points as possible)…………………………………………………………………………….………………………………………………………………………………………………………………………………………………………………………………………………………………………………………………………………………………………………………………........................................................................
